# Genomic Heterogeneity and Clonal Evolution in Gastroesophageal Junction Cancer Revealed by Single Cell DNA Sequencing

**DOI:** 10.3389/fonc.2021.672020

**Published:** 2021-05-11

**Authors:** Qingke Duan, Chao Tang, Zhao Ma, Chuangui Chen, Xiaobin Shang, Jie Yue, Hongjing Jiang, Yan Gao, Bo Xu

**Affiliations:** ^1^ Department of Biochemistry and Molecular Biology, Tianjin Medical University Cancer Institute and Hospital, National Clinical Research Center for Cancer, Key Laboratory of Cancer Prevention and Therapy, Tianjin, China; ^2^ Tianjin’s Clinical Research Center for Cancer, Key Laboratory of Breast Cancer Prevention and Therapy, Tianjin Medical University, Ministry of Education, Tianjin, China; ^3^ State Key Laboratory of Experimental Hematology, Institute of Hematology and Blood Disease Hospital, Chinese Academy of Medical Sciences and Peking Union Medical College, Tianjin, China; ^4^ Department of Minimally Invasive Esophageal Surgery, Tianjin Medical University Cancer Institute and Hospital, Tianjin, China; ^5^ Center for Intelligent Oncology, Chongqing University Cancer Hospital, Chongqing University School of Medicine, Chongqing, China

**Keywords:** single cell sequencing, gastroesophageal junction cancer, genomic heterogeneity, clonal evolution, metastasis

## Abstract

Gastroesophageal junction (GEJ) cancer is a tumor that occurs at the junction of stomach and esophagus anatomically. GEJ cancer frequently metastasizes to lymph nodes, however the heterogeneity and clonal evolution process are unclear. This study is the first of this kind to use single cell DNA sequencing to determine genomic variations and clonal evolution related to lymph node metastasis. Multiple Annealing and Looping Based Amplification Cycles (MALBAC) and bulk exome sequencing were performed to detect single cell copy number variations (CNVs) and single nucleotide variations (SNVs) respectively. Four GEJ cancer patients were enrolled with two (Pt.3, Pt.4) having metastatic lymph nodes. The most common mutation we found happened in the TTN gene, which was reported to be related with the tumor mutation burden in cancers. Significant intra-patient heterogeneity in SNVs and CNVs were found. We identified the SNV subclonal architecture in each tumor. To study the heterogeneity of CNVs, the single cells were sequenced. The number of subclones in the primary tumor was larger than that in lymph nodes, indicating the heterogeneity of primary site was higher. We observed two patterns of multi-station lymph node metastasis: one was skip metastasis and the other was to follow the lymphatic drainage. Taken together, our single cell genomic analysis has revealed the heterogeneity and clonal evolution in GEJ cancer.

## Introduction

Cancers of the upper gastrointestinal tract include esophageal cancer (EC), gastroesophageal junction (GEJ) cancer and gastric cancer (GC). Esophageal cancer is classified as squamous cell carcinoma or adenocarcinoma by histopathology (1), while GEJ cancer and GC are mainly adenocarcinoma (2–4). Although GEJ cancer can be classified as a part of EC or GC, in most cases it is categorized as the latter. However, GEJ cancer and distal GC show different characteristics in epidemiology, risk factors, origin, and prognosis (5, 6). GEJ cancer shows stronger penetrability, more prone to lymph node metastasis and worse prognosis, comparing to GC (7). In recent years, the incidence of GC has gradually decreased owing to the effective eradication of Helicobacter pylori infection, but the incidence of GEJ cancer is gradually increasing attributed to the major risk factor of reflux diseases (8, 9).

Genomic studies on GEJ cancer have mainly focused on bulk sequencing (10–12). For example, in a bulk genomic study on GC patients (including GEJ cancer), the identification of GC subtypes provided the possibility of targeted therapies (10). Another bulk genomic study of large cohort GEJ cancer patients found the independent markers for survival time (11). However, tumor has obvious intra-tumor heterogeneity (ITH). Bulk sequencing can only show the characteristics of major cell populations, missing the heterogeneity information. Single cell sequencing is a powerful tool to resolve ITH (13, 14). The single cell studies on upper gastrointestinal tumors mainly focus on the RNA level at present (15–17). Single cell RNA sequencing can identify the cell composition and transcriptional expression. But it cannot reveal the clonal evolution of lymph node metastasis, which is associated with poor prognosis of cancers. In addition, tumor occurs by acquiring a series of mutations over time (18), and genomic instability and mutation are hallmarks of tumors (19, 20). At present, there is no study on GEJ cancer with metastatic lymph nodes at the single cell genomic level.

Single cell DNA sequencing can be used to reveal ITH and phylogeny at the copy number variation (CNV) and single nucleotide variation (SNV) level, and it has been widely used in cancer study (21–23). Multiple Annealing and Looping Based Amplification Cycles (MALBAC) is a single cell DNA sequencing method, which can significantly reduce amplification bias and improve the genome uniformity (24). In this study, by using the MALBAC method, we are the first to reveal the genomic heterogeneity and clonal evolution of lymph node metastasis in GEJ cancer at single cell level. Our study found significant intra-tumor heterogeneity and distinct patterns of lymph node metastasis in GEJ cancer. These results may improve our understanding of GEJ cancer.

## Materials and Methods

### Sample Collection

Four GEJ cancer patients were enrolled from Tianjin Medical University Cancer Institute and Hospital (from May 2020 to October 2020). Inclusion criteria: (1) treatment naive patients; (2) patients undergoing radical surgery. The information of the GEJ cancer patients was summarized in **Table S1**. All the four patients’ surgical primary tumor tissues and adjacent normal tissues were collected. Three patients were diagnosed with positive lymph nodes. Positive lymph nodes of two patients were also collected (The lymph nodes in one patient were not suited for single cell sequencing). This study was approved by the Ethics Committee of Tianjin Medical University Cancer Institute and Hospital (NO.bc2020180).

### Single Cell Isolation and Genomic DNA Extraction

The acquired GEJ tumor tissues and positive lymph nodes were mechanically dissociated, part of which was used to extract genomic DNA and part was used to isolate single cells. The normal tissues were only used to extract genomic DNA. The genomic DNAs were extracted with QIAamp^®^ DNA Micro Kit (QIAGEN, Hilden, Germany) according to the manufacturer’s protocol.

For single cell isolation, the tissues were digested with tumor dissociation kit (Miltenyi Biotec, Bergisch Gladbach, Germany). Erythrocytes were depleted by red blood cell lysis buffer (Solarbio, Beijing, China). Leukocytes were removed with Dynabeads™ CD45 (Invitrogen, Waltham, MA, USA). The single cells were isolated with mouth pipetting and transferred to tubes containing the lysis buffer (0.02 M Tris-HCl, 2 mM EDTA, 0.02 M KCl, 0.015 M DTT, 0.25 μM 5N3G Primer, 5 μg QIAGEN protease). The cells were lysed and stored at −80°C until amplification.

### Single Cell Whole Genome Amplification

Single cell amplification was carried out according to the previously reported MALBAC method (24). This whole genome amplification method introduced quasi-linear preamplification, which could reduce the amplification bias. In brief, nine cycle preamplification and subsequent exponential amplification were performed. The amplification products were purified with AMPure XP beads (Beckman Coulter, Brea, CA, USA) and quantified with Qubit 4.0 Fluorometer (Invitrogen, Waltham, MA, USA). Quantitative PCR (qPCR) was used to check the integrity of the genome (the sequences of qPCR primers were shown in **Table S2**). Cells having no less than six out of eight randomly selected loci with Ct value<30 could be used for library preparation and next generation sequencing.

### Library Preparation and Sequencing

For single cells, we constructed the whole genome library for each cell. The selected single cell DNAs were sonicated with Covaris S220 to produce short DNA fragments. Then, libraries were prepared with KAPA Hyper Prep Kit (KAPA, Boston, MA, USA) and NEBNext Multiplex Oligos for Illumina (NEB, Ipswich, MA, USA) according to manufacturer’s protocol. Briefly, main steps included end repair and A-tailing, adapter ligation, post-ligation cleanup and library amplification. qPCR and fragment analysis were used for library quality control. The selected libraries were sequenced with Illumina NovaSeq 6000.

The exome libraries were constructed with Agilent SureSelect Human All Exon V6 kit (Agilent Technologies, Santa Clara, CA, USA) according to manufacturer’s protocol. Briefly, the genomic DNA was sonicated with Covaris S220 to produce short DNA fragments. After end repair with exonuclease/polymerase, A-tailing in 3’ ends of DNA fragments and adapter ligation, the DNA fragments with adapter at both ends were enriched by PCR. The biotin labeled probe was used to hybridize with DNA libraries and the exons were captured with magnetic beads and streptomycin. Then, the captured libraries were added index tags and enriched by PCR reaction. qPCR and fragment analysis were used for library quality control. The selected libraries were sequenced with Illumina NovaSeq 6000. The sequencing data has been submitted to NCBI SRA under BioProject accession number PRJNA718709.

### CNV and CNV Frequency Estimation From Single Cell DNA Sequencing Data

First, the raw sequencing reads were trimmed to remove adaptors and low quality bases using Trimmomatic (25). Pair-End mode with ‘HEADCROP:35’ was used to remove the low quality bases from the start of the read and other default parameters. Then, the clean reads were mapped to the human reference genome hg38 with Burrows–Wheeler Alignment tool (BWA) using ‘MEM’ command in Paired-End mode and unmapped reads were realigned to the same reference genome in Single-End mode (26). The PCR duplicates were removed using SAMtools with ‘markdup’ (27). After alignment, the single cell copy number values were calculated with Control-FREEC at a resolution of 2M genomic window size (28). The raw copy number matrices obtained from Control-FREEC were processed in R software. First, copy number values less than one were converted to one, and the copy number values greater than four were converted to four. Then, the copy number values were log2 transformed and they were centered to zero by subtracting one for each cell.

Frequencies of CNVs in primary GEJ cancer were calculated based on the copy number value matrix after preliminary transformed. With a window size of 2M, the values greater than 0.3 were regarded as copy number gains while less than -0.3 were regarded as copy number losses. The number of cells with gains or losses in each window were divided by the total primary tumor cell number respective to each patient to eliminate effect of the unbalanced cell numbers among the four patients. Then the four weighted values of each window were summed together and divided by four.

### CNV Clustering and Clonal Evolution Analysis

To identify the number of clusters, Euclidean distances between cells were calculated and ‘hclust’ was used to perform hierarchical clustering. To delineate the clonal evolution during invasion, the genomic lineages were inferred, and the data were plotted using R package Timescape (29). The clones were defined according to the clustering results and the proportions of different origins of cells that belonged to each clone were calculated.

### SNV Analysis From Bulk Exome Sequencing

Quality control of the raw sequencing data was performed by discarding reads containing adapter contamination, low-quality nucleotides, and unrecognizable nucleotides. Then the clean reads were mapped to the UCSC human reference genome by BWA-MEM algorithm (26). If a read was mapped to multiple positions, BWA allowed to choose the most likely placement (if the multiple placements were most likely, the choice was random). SAMtools was used to sort and merge the mapped reads and Picard was used for duplicate removal. After removing the duplicate reads, recalibrating the base quality scores and local realignment (30), Samtools mpileup and bcftools were used to do variant calling and the somatic SNVs were detected by muTect, which was based on a Bayesian classifier (31). The variants were determined by analyzing the LOD score and the systematic false positive was decreased with the use of filters. The functional annotation of variants, including information in gene transcript annotation databases, such as Consensus CDS, RefSeq, Ensembl and UCSC, and other related databases, such as dbSNP and 1000 Genome, was given using ANNOVAR (32).

### Phylogenetic Analysis

The phylogenetic trees were constructed based on the distribution of nonsynonymous SNVs by MEGA5 software. The max-mini branch-and-bound algorithm was used to infer the maximum-parsimony trees (33). We used the average pathway method (34), to calculate branch lengths. The normal tissues were set as the outgroup to gain the consensus tree. Then, the phylogenetic trees were redrawn in Adobe Illustrator software. The lengths of trunk and branch were proportional to the acquired average number of nonsynonymous mutations, with the terminal branches colored in red, the internal branches colored in yellow, and the trunk colored in blue. The angles between branches were chosen just for convenient display. Representative driver SNVs were marked next to the corresponding branches.

## Results

### Mutation Landscapes of the GEJ Cancer Patients

**Figure 1:**
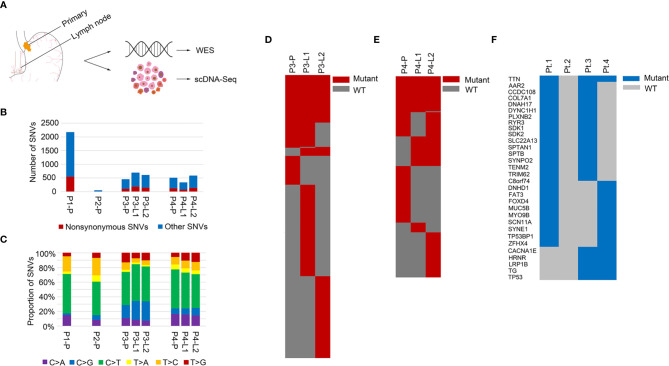
Mutation landscapes of the GEJ cancer patients. **(A)** Overview of the project: genomic profiling of primary tumor and lymph node metastases in the GEJ cancer patients. WES: whole exome sequencing. **(B)** The number of somatic mutations was shown. X axis represented different samples and Y axis represented the number of SNVs. **(C)** Mutation spectra of primary and lymph node sites were shown. X axis represented different samples, Y axis represented the proportion of SNVs, and different colors represented different mutation types. **(D)** A heatmap showed the distribution of nonsynonymous SNVs of primary and lymph nodes in Pt.3. **(E)** A heatmap showed the distribution of nonsynonymous SNVs of primary and lymph nodes in Pt.4. **(F)** A heatmap showed the recurrent mutated genes shared in at least two of the four GEJ cancer patients.

In this study, four patients were enrolled. We obtained the primary tumors from all the patients and also two lymph nodes from two of the patients (Pt.3, Pt.4) respectively. For Pt.3 and Pt.4, L1 represented the lymph node anatomically closer to the primary tumor compared to L2. The sample information and clinical characteristics of the four GEJ cancer patients were shown in **Table S1**. In order to better understand the genomic heterogeneity and phylogeny of GEJ cancers, we first performed whole exome sequencing (WES) on primary tumor and metastatic lymph node samples to analyze the mutations. The overview of project was shown in **Figure 1A**.

The somatic SNVs of the primary tumor and lymph nodes were called using the matched normal tissue samples as control. The mutation numbers of the four GEJ cancer patients were shown in **Figure 1B**. For the primary sites, Pt.1 had the largest number of both nonsynonymous SNVs and total SNVs. As was known, mutations accumulated as age increased and Pt.1 was the oldest among the four patients. The number of SNVs in Pt.2 was the least. The primary tumor in Pt.2 was large, but no clinical metastasis was diagnosed, which might be related to less tumor mutation burden. We also analyzed the mutation spectra, showing similar mutation types among the four patients (**Figure 1C**). Whether in the primary or lymph node sites, the C > T mutation was significantly enriched, consistent with the results of a relatively large set of Chinese GEJ cancer patients (11).

Although there were similar mutation spectra among patients, the numbers of mutations varied among patients or different sampled sites in individuals. **Figures 1D, E** showed the common SNVs and the primary or lymph node specific SNVs in Pt.3 and Pt.4. The proportion of primary or lymph node specific mutation (the number of primary or lymph node mutations divided by the common mutations) were distinct between Pt.3 (0.40, 2.49) and Pt.4 (0.94, 1.40). Also, numbers of primary tumor and lymph node specific mutations were distinct within the same patient. In Pt.3, the lymph node specific SNVs were significantly more than the primary site (6.31-fold). In Pt.4, the lymph node specific SNVs versus primary site was 1.49-fold.

Next, the recurrent mutated genes among the four patients were analyzed. **Figure 1F** showed that the most frequently mutated gene was TTN, which occurred in three patients. Eight mutation sites were identified, six of which occurred in Pt.4. The mutation sites of TTN gene were shown in **Figure S1**. Most of the mutation sites of TTN occurred in the immunoglobulin I-set domain. Other frequent mutations were in TP53, TG, LRP1B, AAR2, CCDC108, COL7A1, DNAH17, DYNC1H1, PLXNB2, etc., which happened in two patients.

### SNV Subclonal Architecture Between Lymph Nodes

The mutation allele frequency was used to identify the clonal or subclonal mutations between different sampled sites (35). For Pt.1 and Pt.2 that only had primary tumors, the mutation frequencies of nonsynonymous SNVs were shown in **Figure S2**. Almost all the SNVs were subclonal, indicated the intra-tumor heterogeneity. Next, we analyzed the mutation allele frequency between primary tumor and matched lymph nodes (**Figure S3**). **Figures S3A, B** showed that the mutation subclonal architecture was relatively more conserved between the primary tumor and L1 than primary tumor and L2 in Pt.3. While the subclonal structures between primary tumor and L1 were similar to that between primary tumor and L2 in Pt.4 (**Figures S3C, D**).

**Figure 2:**
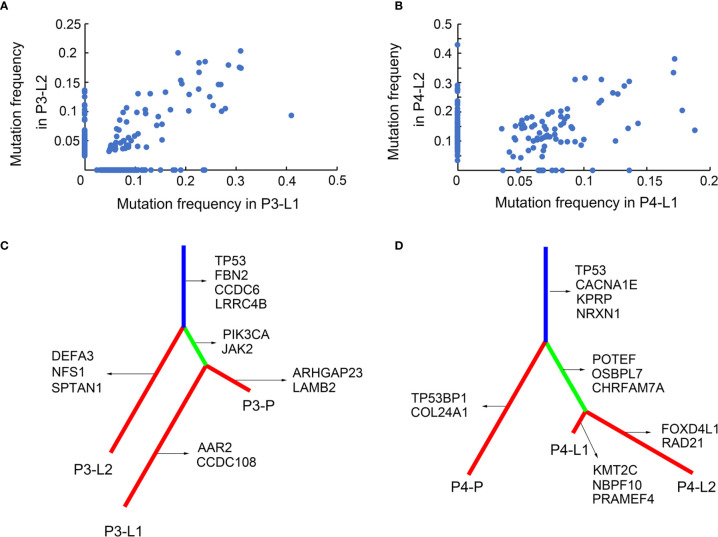
SNV subclonal architecture and phylogenetic analysis. **(A)** The mutation frequencies of nonsynonymous SNVs were shown in two different LN sites in Pt.3. **(B)** The mutation frequencies of nonsynonymous SNVs were shown in two different LN sites in Pt.4. **(C)** A phylogenetic tree based on SNVs in Pt.3. The genes with inferred driver mutations corresponding to individual branches and trunk were indicated. **(D)** A phylogenetic tree based on SNVs in Pt.4.

For Pt.3 and Pt.4 who had two matched lymph nodes, the mutation frequencies were analyzed between the lymph nodes. **Figures 2A, B** showed that the mutation frequencies between the two lymph nodes in Pt.4 were more similar than that in Pt.3, suggesting that the subclonal structures of SNVs were relatively more conserved between the two lymph nodes in Pt.4.

### SNV-Phylogenetic Analysis of Primary Tumor and Lymph Nodes

The above results revealed the mutation profiles and subclonal architectures of the four GEJ cancer patients. However, the lymph node metastasis patterns of different patients still needed further analysis. The SNV based phylogenetic trees in Pt.3 and Pt.4 with lymph node metastasis were constructed. **Figure 2C** showed that the two lymph nodes of Pt.3 were in different branches, while one of the lymph nodes and primary tumor were on the same branch, indicating that the two lymph nodes metastasized independently. The distant lymph node (L2) branched from the trunk earlier, indicating that L2 metastasized earlier than L1. However, the phylogeny pattern of Pt.4 showed that the primary site was branched from the trunk, and the two lymph nodes were separated from another branch (**Figure 2D**). **Figure 2D** indicated that the two lymph nodes had the same genomic ancestor descended from the primary site and metastasized following the lymphatic drainage.

The genes included in COSMIC (Catalogue Of Somatic Mutations In Cancer) database and KEGG pathways in cancer were defined as the inferred driver mutations and marked in the phylogenetic trees. Mutation of TP53 was trunk mutation in both Pt.3 and Pt.4, indicating that TP53 mutations occurred earlier and played an important role in tumor progression and lymph node metastasis. For Pt.3, PIK3CA, JAK2 etc. mutations occurred only in L1, not in L2. This might be related to the phenomenon that the two lymph nodes were separated different branches from the trunk. While in Pt.4, the two lymph nodes were separated from the same branches, simultaneously showing the mutation of POTEF, OSBPL7 and CHRFAM7A.

The SNV-phylogenetic analysis revealed distinct phylogeny patterns of lymph node metastasis in the two patients. The lymph nodes could separate independently from the primary tumor at different time points, and also metastasize station by station following the lymphatic drainage.

### CNV Heterogeneity of Primary Tumor and Lymph Nodes

**Figure 3:**
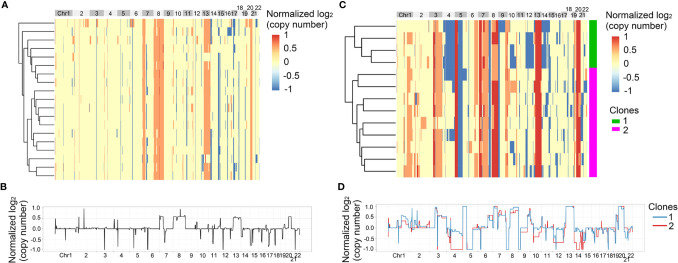
CNV heterogeneity of primary tumor in Pt.1 and Pt.2. **(A)** A heatmap of single cell copy number profiles in Pt.1 with the clustering analysis based on CNVs on the left. **(B)** A plot showed the normalized average copy numbers of all the single cells from primary tumor in Pt.1. **(C)** A heatmap of single cell copy number profiles in Pt.2 with the clustering analysis based on CNVs on the left. All the cells were divided into two subclones. **(D)** A plot showed the normalized average copy numbers of the two subclones from primary tumor in Pt.2.

In the above results, we found that SNVs were heterogeneous between primary tumor and matched metastatic lymph nodes. Next, we analyzed the heterogeneity of CNVs at single cell level, which could not be found by bulk sequencing. 210 single cells from the four patients passed quality control and the single cell information was shown in **Table S3**. First, we analyzed the heterogeneity of Pt.1 and Pt.2, who only had the primary tumors collected. The heatmap in **Figure 3A** showed the global copy number profiles in Pt.1. The data identified the CNV events to be clonal, due to the similar CNV patterns of all the single cells. **Figure 3B** showed the normalized CNVs, including the amplification in Chr8, 13, 20 and deletion in Chr14, 15. The heatmap in **Figure 3C** showed the global CNV profiles in Pt.2. The data identified two major clones in the primary tumor. **Figure 3D** showed the CNV patterns of the two subclones. The shared CNVs included the amplification in Chr7, 13, 20 and deletion in Chr5q. There were also distinct CNV regions between the two subclones, such as a greater degree of deletion on Chr4 and 14 in subclone 2 compared to subclone 1.

**Figure 4:**
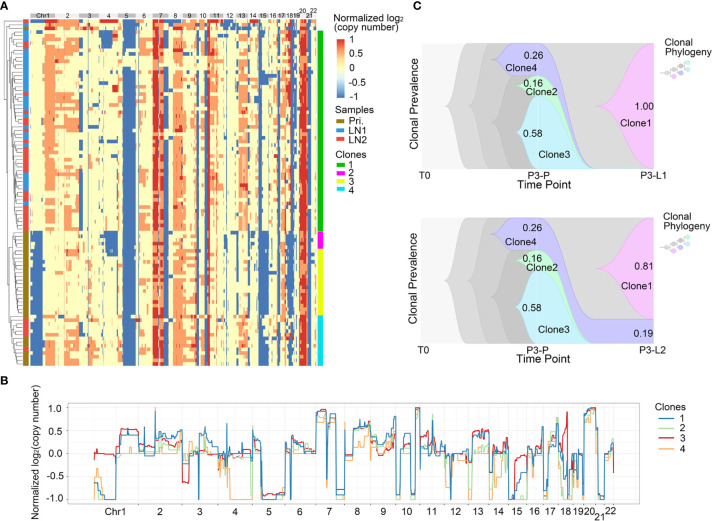
CNV heterogeneity and clonal evolution analysis of primary tumor and lymph nodes in Pt.3. **(A)** A heatmap of single cell copy number profiles in Pt.3 with the clustering analysis based on CNVs on the left. All the cells were divided into four subclones. **(B)** A plot showed the normalized average copy numbers of the four subclones in Pt.3. **(C)** The clonal evolution from primary to L1 (upper) and primary to L2 (lower) with the clonal prevalence changes indicated in Pt.3.

**Figure 5:**
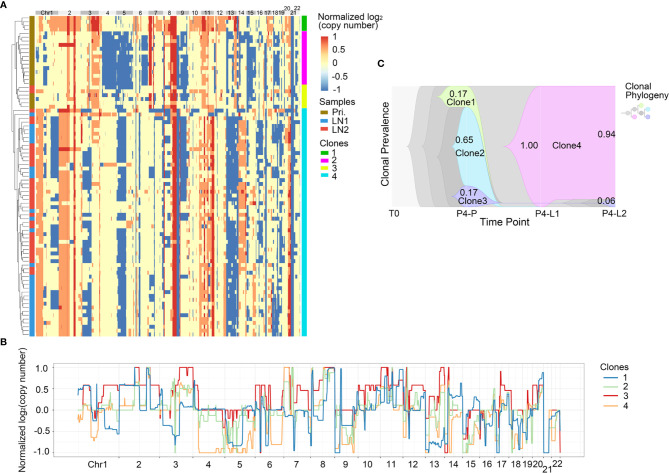
CNV heterogeneity and clonal evolution analysis of primary tumor and lymph nodes in Pt.4. **(A)** A heatmap of single cell copy number profiles in Pt.4 with the clustering analysis based on CNVs on the left. All the cells were divided into four subclones. **(B)** A plot showed the normalized average copy numbers of the four subclones in Pt.4. **(C)** The clonal evolution from primary to L1 and L2 with the clonal prevalence changes indicated in Pt.4.

Next, we analyzed the intra-patient heterogeneity in Pt.3 and Pt.4, who had the single cell information of the primary tumor and lymph nodes. **Figures 4A**, **5A** showed the global CNV profiles of the individual cells in Pt.3 and Pt.4 respectively. The data identified four major clones in each patient. **Figure 4B** showed the CNV patterns of the four subclones in Pt.3. The shared CNVs included the amplification in Chr7p,20 and deletion in Chr5q,21. The subclone specific CNV events were also observed. **Figure 5B** showed the CNV patterns of the four subclones in Pt.4. The differences of CNV profiles between subclones revealed obvious intra-patient heterogeneity. This heterogeneity not only existed in the primary tumor or lymph nodes, but also existed between the primary tumor and lymph nodes.

It was reported that CNV events in primary tumor often also occurred in single or multiple matched lymph nodes, but a few CNVs in the lymph nodes were not found in primary tumor (36). This indicated that the degree of heterogeneity between primary tumor and lymph nodes were different. **Figure 4A** revealed that in Pt.3 the primary tumor consisted of three subclones, while the L1 and L2 lymph nodes consisted of one or two subclones. **Figure 5A** also revealed that in Pt.4 the primary tumor consisted of three subclones, while the L1 and L2 lymph nodes consisted of one or two subclones. The results indicated that the heterogeneity of primary tumor was higher than that of lymph nodes in the same patient.

### CNV-Clonal Evolution Analysis

Unlike the evolutionary model of mutation, which evolved gradually, resulting in extensive clonal diversity, the CNVs occurred in the early stage of tumor evolution and were highly stable with the clonal expansion of tumor (13). Our above mutation data showed that the phylogeny patterns of lymph node metastasis in Pt.3 and Pt.4 were distinctive (**Figure 2**). Next, we analyzed the modes of clonal evolution using CNV data. The clonal frequencies could be analyzed to visualize the clonal changes with tumor metastasis (29). Based on previous SNV data, the two lymph nodes in Pt.3 originated from the primary tumor independently, so we analyzed the proportion changes of each subclone from primary to L1 and L2 lymph nodes separately.

In Pt.3, there were three subclones in primary tumor, including clone 2, clone 3 and clone 4 with percentages of 16%, 58% and 26% (**Figure 4C**). In the lymph nodes, the clone 3, which was the main proportion of primary tumors, and clone 2 disappeared. However, a new subclone (clone 1) appeared and accounted for 100% in L1 lymph node. Two subclones (clone 1 and clone 4) were found in L2 lymph node and clone 1 accounted for 81%. The main differences of CNVs between clone 3 and clone 1 were clone 1-specific deletion in Chr1p and 18. These CNVs might be related to lymph node metastasis in Pt.3.

In Pt.4, there were three subclones in the primary tumor, including clone 1, clone 2 and clone 3 with percentages of 17%, 65% and 17% (**Figure 5C**). The clone 1 and clone 2 disappeared in the lymph nodes. However, a new subclone (clone 4) appeared in the lymph nodes and accounted for 100% in L1 lymph node and 94% in L2 lymph node. The main differences of CNVs between clone 4 and other subclones were that the obvious deletion of Chr4 and 6. These CNVs might be related to lymph node metastasis in Pt.4.

The clonal evolution analysis in Pt.3 and Pt.4 showed that the proportion of different subclones changed significantly with the metastasis of GEJ cancer. The findings further revealed the molecular mechanism of tumor metastasis in GEJ cancer.

## Discussion

The mutation spectra in our study revealed that C > T mutations were significantly enriched in the patients whether in the primary site or lymph node site, indicating that C > T mutation played a crucial role in GEJ tumorigenesis and progression. This was consistent with other studies (6, 37, 38). Our study identified the recurrent mutations happened in TTN, TP53, AAR2, CCDC108, CACNA1E, RYR3, LRP1B etc. genes. Among these mutation genes, TP53 was also reported as the most significantly mutated gene in a recent study of 124 Chinese GEJ cancer patients (11). Another study focusing on 12 cases of synchronous GEJ cancer and distal gastric cancer revealed the predominance of TP53 mutation (6). The recurrent mutations of TTN, TP53, RYR3 and LRP1B genes of this study were also found in our study, but we identified new frequently mutated genes, such as AAR2, CCDC108 and CACNA1E genes.

Considering that the anatomical position of GEJ cancer was between stomach and esophagus, we compared both our CNV and SNV results of the four GEJ cancer patients with data of gastric and esophagus by TCGA (10, 39). For CNVs, we normalized different cell numbers among patients and calculated CNV frequencies based on 86 single cells from primary tumors of our four GEJ cancer patients, shown in **Figure S4**. Compared with TCGA data in gastric and esophagus cancers, similarities were observed among GEJ cancer and EAC (esophagus adenocarcinoma) or GA-CIN (gastric adenocarcinoma-chromosomal instability), such as high frequencies of copy number gains in Chr7, 8, 20 and losses in Chr4, 5, 18, 21. For SNVs, we compared the genes mutated in at least two of the four GEJ cancer patients with top mutated genes listed in the two papers. Only one mutated gene was shared by GEJ cancer and gastric or esophagus cancer: TP53, exhibiting distinct mutation landscapes of them. And the mutation frequency of TP53 in our GEJ cancer patients was 50%, much lower than that in EC [71% in EAC and 91% in ESCC (esophagus squamous cell carcinoma)], but close to that in GC (50% in non-hypermutated gastric cancer and 35% in hypermutated gastric cancer).

In our study, we identified that TTN was the most frequently mutated gene. The function of TTN (Titin) was mainly studied in the muscle contraction (40, 41). There were also reports that TTN was at the top ranking of mutated genes in multiple solid tumors, including the gastric adenocarcinoma, small cell lung cancer and colorectal adenocarcinoma (42). The average mutation frequency of TTN in solid tumors was 29.68% and the mutation frequency in gastric adenocarcinoma was as high as 60%~70% (42). The mutation frequencies of TTN gene could also be used to represent the tumor mutation burden (43). In a study of 12 patients with synchronous GEJ cancer and GC, the TTN mutation were found in 7 of 24 tumor tissues (6). However, the mechanism of TTN in tumorigenesis and progression remained unclear and was worthy of further study. Besides, we further followed the previous study (11) to find CNVs/SNVs predicting vulnerabilities to chemotherapeutic or targeted therapeutic agents approved by FDA. We listed these CNVs and SNVs in **Table S4**. Among them were mutations in ARID1A, ATM, BRCA1 and TP53 genes, amplifications in CCND1, ERBB2 and MYC genes, deletions in PTEN and RB1 genes etc.

The phylogenetic patterns of lymph nodes in Pt.3 and Pt.4 existed obvious differences. In Pt.3, the similarity between lymph nodes and primary tumor was greater than that between the different lymph nodes, indicating that the two lymph nodes both arose from the primary tumor and they were independently metastasized. The phylogenetic tree of Pt.3 revealed that the L2 lymph node separated from the trunk very early. As the L2 lymph node represented the relatively distant lymph node anatomically, this pattern of clonal evolution indicated the independent and skip metastasis in Pt.3. The two lymph nodes of Pt.4 were in the same branch separated from the primary site, indicating that the metastasis was following the lymphatic drainage in Pt.4. GEJ cancer patients in China were mainly treated as gastric cancer, as Chinese esophageal cancer patients were mainly squamous cell carcinoma (44). For this reason, we compared the evolutionary models between our study and reported gastric cancer studies. Unlike our study, previous study on gastric cancer revealed a common phylogeny pattern in which all the three lymph nodes appeared in a single branched cluster (45).

We also analyzed the CNV data at single cell level. Previous study showed that 20-40 single cells in each patient were necessary to study the major subclones with 95% power (46). Therefore, the single cells in our study were sufficient to describe the subpopulation composition. The single cell copy number profiles and subclonal compositions showed that there was obvious heterogeneity in GEJ cancer patients. The subclonal composition of primary tumor was more complex than that of lymph nodes in both Pt.3 and Pt.4 patients, indicating that the heterogeneity of primary site was significantly higher than that of lymph nodes. This might imply that the CNVs play a decisive role in tumor metastasis and that only cells with specific CNV patterns have the ability of metastasis, which is consistent with previous studies (47).

Comparing to the primary subclones, new subclones were found in lymph nodes in Pt.3 and Pt.4. The L1 lymph nodes were formed entirely by a new subclone, and L2 lymph nodes contained two subclones: one subclone from the primary site and the other a new subclone. The possible reason for the new subclones in the lymph nodes might be that the subclone in the primary site was transferred to lymph nodes entirely, or there was limited single cells sequenced in the primary tumor. Our study did not compare the single cell CNVs between GEJ cancer with GC because there was no single cell CNV data for GC so far.

Our study has several advantages. First, single cell genomic sequencing method is used to study the tumor heterogeneity and clonal evolution of lymph node metastasis in GEJ cancer for the first time. Second, the SNVs and CNVs are combined for analysis to reveal the possible mechanisms of lymph node metastasis. It should be noted that this study has several limitations. For example, the limited number of patients and single cells that are analyzed. Further studies will expand the number of GEJ cancer patients and single cells in each patient. In addition, validating the mechanistic roles of the driver mutation genes are needed.

In conclusion, our study reveals the SNVs and single cell CNV profiles in GEJ cancer patients. The patterns of lymph node metastasis are revealed from both SNV and CNV levels. These findings will help us better understand the genomic variations and mechanisms of lymph node metastasis.

## Data Availability Statement

The sequencing data has been submitted to NCBI SRA under BioProject accession number PRJNA718709.

## Ethics Statement

The studies involving human participants were reviewed and approved by Ethics Committee of Tianjin Medical University Cancer Institute and Hospital. The patients/participants provided their written informed consent to participate in this study.

## Author Contributions

QD, YG, and BX designed this study. ZM, CC, XS, JY, and HJ provided the tissue specimens and clinical information. QD and YG performed the experiments. QD, CT, and YG acquired and analyzed the data. QD, YG, and BX wrote the manuscript. All authors contributed to the article and approved the submitted version.

## Funding

This work was supported by Beijing Tianjin Hebei Basic Research Cooperation Project (No.: 19JCZDJC64500(Z)) and National Natural Science Foundation of China (No.: 81974464, 81903124, and 81672743).

## Conflict of Interest

The authors declare that the research was conducted in the absence of any commercial or financial relationships that could be construed as a potential conflict of interest.
