# Supplementary material for: Genomic Heterogeneity and Clonal Evolution in Gastroesophageal Junction Cancer Revealed by Single Cell DNA Sequencing
Source: Front Oncol. 2021 May 11;11:672020. doi: 10.3389/fonc.2021.672020 (PMC8144650; doi:10.3389/fonc.2021.672020)
Supplement: Supplementary file 1 [file DataSheet_1.pdf]

# Supplementary Material

## 1 Supplementary Figures and Tables

### 1.1 Supplementary Figures

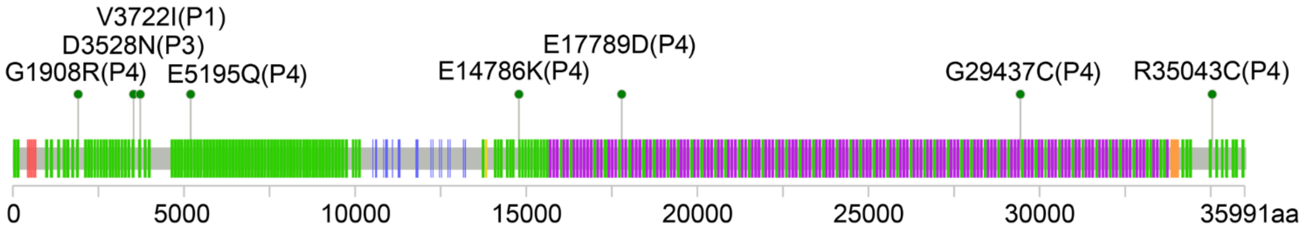


**Supplementary Figure 1.** TTN mutations. Interpretation of recurrent TTN mutations in the GEJ cancer patients with protein annotations. The patient numbers were marked in the brackets.

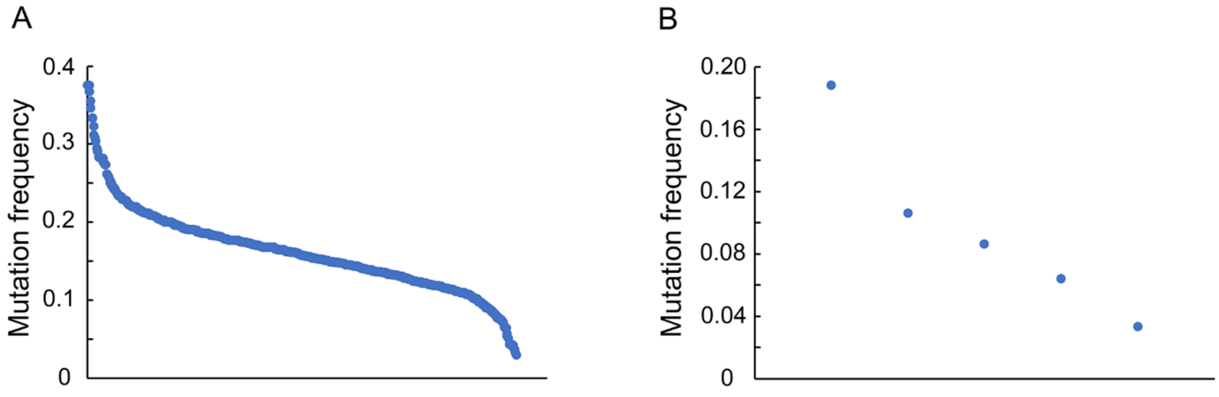


**Supplementary Figure 2.** SNV subclonal architecture of primary tumor in Pt.1 and Pt.2. **(A)** The mutation frequencies of nonsynonymous SNVs in primary tumor were shown in Pt.1. **(B)** The mutation frequencies of nonsynonymous SNVs in primary tumor were shown in Pt.2.

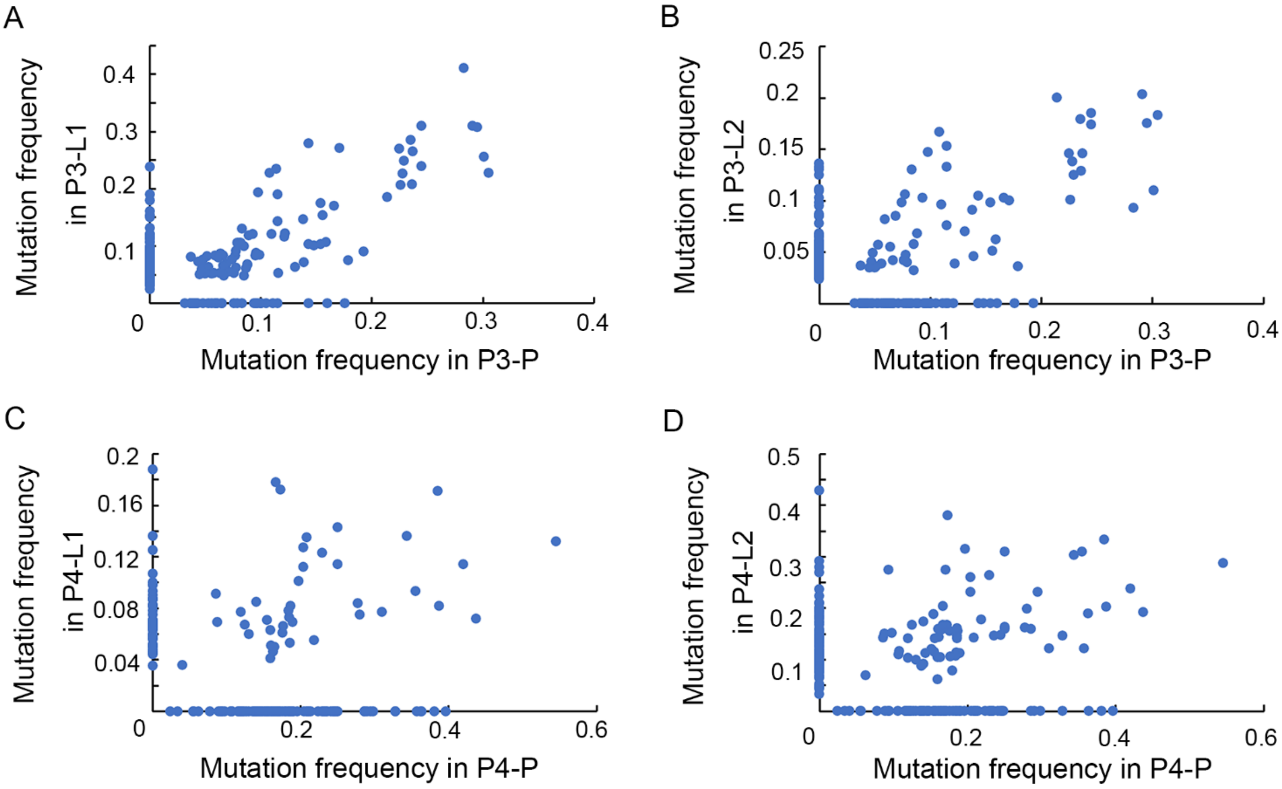


**Supplementary Figure 3.** SNV subclonal architecture between primary tumor and lymph nodes. **(A)** The mutation frequencies of nonsynonymous SNVs were shown in primary tumor and L1 lymph node in Pt.3. **(B)** The mutation frequencies of nonsynonymous SNVs were shown in primary tumor and L2 lymph node in Pt.3. **(C)** The mutation frequencies of nonsynonymous SNVs were shown in primary tumor and L1 lymph node in Pt.4. **(D)** The mutation frequencies of nonsynonymous SNVs were shown in primary tumor and L2 lymph node in Pt.4.

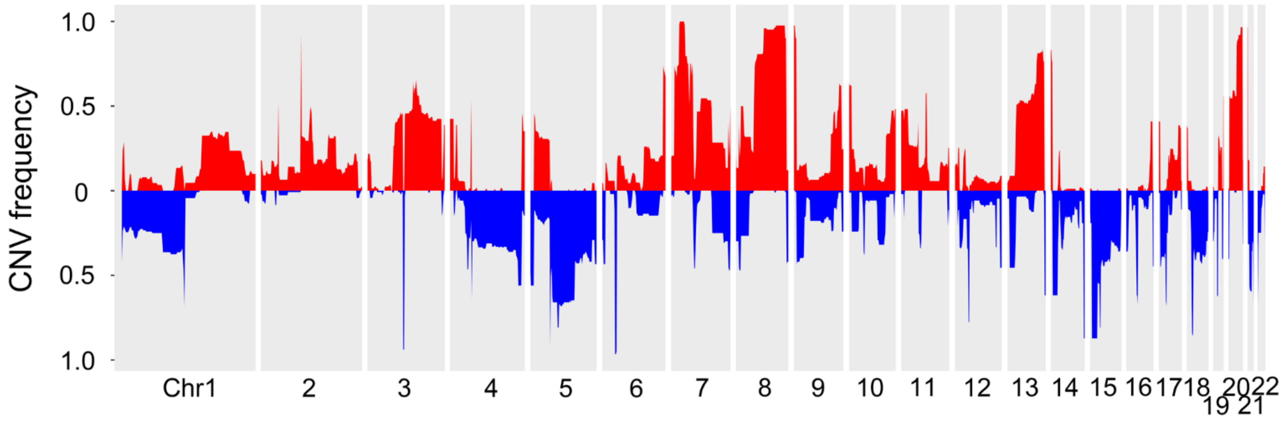


**Supplementary Figure 4.** Frequencies of CNVs detected in GEJ cancer patients. We normalized different cell numbers among patients and calculated CNV frequencies based on 86 single cells from primary tumors of the four GEJ cancer patients in 2M window size, with gains in red and losses in blue.

## 1.2 Supplementary Tables

**Supplementary Table 1** Sample information and clinical characteristics of GEJ cancer patients

| | Pt.1 | Pt.2 | Pt.3 | Pt.4 |
|---|---|---|---|---|
| Gender | Female | Male | Male | Male |
| Age | 82 | 64 | 67 | 59 |
| AJCC Stage | IIB | IIB | IIIA | IIIA |
| Differentiation | Moderate | Moderate | Moderate | Moderate |
| Pathological type | Adenocarcinoma | Adenocarcinoma | Adenocarcinoma | Adenocarcinoma |
| Normal adjacent tissue | √ | √ | √ | √ |
| Primary tumor | √ | √ | √ | √ |
| Diagnosed lymph nodes | 3/26* | 0/44 | 9/26 | 8/19 |
| Sequenced lymph nodes | 0 | 0 | 2 | 2 |

√ : were present and analyzed in this study

* A/B: A represented the number of positive lymph nodes in pathological diagnosis; B represented the number of lymph nodes sent for pathological diagnosis.

**Supplementary Table 2** Sequences of qPCR primers

| Primer name | | Sequences |
|---|---|---|
| Chr1 | Forward | AGGAAAGGCATACTGGAGGGACAT |
| | Reverse | TTAGGGATGGCACCACACTCTTGA |
| Chr2 | Forward | TCCCAGAGAAGCATCCTCCATGTT |
| | Reverse | CACCACACTGCCTCAAATGTTGCT |
| Chr3 | Forward | TCAAGTTGCCAGCTGTGGCTGTAT |
| | Reverse | AGAAGGGCATTTCCTGTCAGTGGA |
| Chr4 | Forward | ATGGGCAAATCCAGAAGAGTCCAG |
| | Reverse | CCATTCACTTCCTTGGAAAGGTAGCC |
| Chr5 | Forward | AATAGCGTGCAGTTCTGGGTAGCA |
| | Reverse | TTCACATCCTGGGAGGAACAGCAT |
| Chr6 | Forward | TGAATGCCAGGGTGAGACCTTTGA |
| | Reverse | TGTTCATTATCCCACGCCAGGACT |
| Chr7 | Forward | ACCAAAGGAAAGCCAGCCAGTCTA |
| | Reverse | ACTCCACAGCTCCCAAGCATACAA |
| Chr9 | Forward | TCCCAGCTCTCTCTCTTGCATCTT |
| | Reverse | AGTGAAGCTGGTGTATGCAGAGGT |

**Supplementary Table 3** The number of single cells passed quality control in GEJ cancer patients

| Patient ID | Sampled sites | | | |
|---|---|---|---|---|
| | Primary tumor | Lymph node 1 | Lymph node 2 | Total |
| Pt.1 | 19 | 0 | 0 | 19 |
| Pt.2 | 13 | 0 | 0 | 13 |
| Pt.3 | 32 | 31 | 32 | 95 |
| Pt.4 | 22 | 29 | 32 | 83 |
| Total | | | | 210 |

**Supplementary Table 4** CNVs/SNVs predicting vulnerabilities to chemotherapeutic or targeted therapeutic agents approved by FDA

| SNVs/CNVs | Patient(s) with this alteration | Chemothrapeutic/Targeted therapeutic agents |
|---|---|---|
| ATM Mutation | Pt.1 | Temozolomide, Platinum |
| BRCA1 Mutation | Pt.1 | Platinum |
| MYC Amplification | Pt.1, Pt.2, Pt.3 and Pt.4 | Temozolomide |
| NF1 Mutation | Pt.3 | Vinblastine |
| RB1 Deletion | Pt.4 | Platinum |
| TOP2A Amplification | Pt.3 and Pt.4 | Anthracyclines |
| TP53 Mutation | Pt.3 and Pt.4 | Anthracyclines, Gemcitabine, Mytomycin C |
| ARID1A Mutation | Pt.1 | PARP inhibitor |
| ATM Mutation | Pt.1 | PARP inhibitor |
| CCND1 Amplification | Pt.3 and Pt.4 | CDK4/6 inhibitor |
| CCND3 Amplification | Pt.2, Pt.3 and Pt.4 | CDK4/6 inhibitor |
| CCNE1 Amplification | Pt.1, Pt.2 and Pt.4 | CDK2 inhibitor |
| CDK4 Amplification | Pt.4 | CDK4/6 inhibitor |
| CDK6 Amplification | Pt.2, Pt.3 and Pt.4 | CDK4/6 inhibitor |
| CDKN2A Deletion | Pt.2 and Pt.4 | CDK4/6 inhibitor |
| CDKN2B Deletion | Pt.2 and Pt.4 | CDK4/6 inhibitor |
| ERBB2 Amplification | Pt.3 and Pt.4 | ERBB2 inhibitor |
| FGF3 Amplification | Pt.3 and Pt.4 | FGFR inhibitor |
| FGF4 Amplification | Pt.3 and Pt.4 | FGFR inhibitor |
| FGFR2 Amplification | Pt.3 and Pt.4 | FGFR inhibitor |
| PTEN Deletion | Pt.2 and Pt.3 | PARP inhibitor |

| | | |
|---|---|---|
| TP53 Mutation | Pt.3 and Pt.4 | WEE1 inhibitor |
